# Study protocol: intervention in maternal perception of preschoolers’ weight among Mexican and Mexican-American mothers

**DOI:** 10.1186/s12889-018-5536-0

**Published:** 2018-05-30

**Authors:** Yolanda Flores-Peña, Meizi He, Erica T. Sosa, Hermelinda Avila-Alpirez, Perla M. Trejo-Ortiz

**Affiliations:** 1 Autonomous University of Nuevo Leon (UANL), College of Nursing, Av. Gonzalitos No. 1500 Norte, Col. Mitras Centro, C.P. 64460 Monterrey, Nuevo León Mexico; 2 The University of Texas at San Antonio, College of Education and Human Development, San Antonio, 78249 Texas USA; 3 Autonomous University of Tamaulipas (UAT), College of Multidisciplinary Knowledge, Av, del Maestro y Marte S/N, H. Matamoros, 87410 Tamaulipas Mexico; 4 University of Zacatecas, Academic Unit of Nursing, Carretera Zacatecas - Guadalajara, Km. 6, Ejido la Escondida, Zacatecas, 98160 Zacatecas Mexico

**Keywords:** Multicenter study, Health behavior, Maternal perception, Body weight, Pediatric obesity, Mother-child relations, Parenting

## Abstract

**Background:**

Childhood obesity is a public health issue negatively affecting children’s physical and psychosocial health. Mothers are children’s primary caregivers, thus key players in childhood obesity prevention. Studies have indicated that mothers underestimate their children’s weight. If mothers are unaware of their children’s weight problem, they are less likely to participate in activities preventing and treating excess weight. The “Healthy Change” intervention is designed to change maternal perception of child’s weight (MPCW) through peer-led group health education in childcare settings.

**Methods/Design:**

The “Healthy Change” is a multicenter two-arm randomized trial in four centers. Three centers are in Mexican States (Nuevo Leon, Tamaulipas, and Zacatecas). The fourth center is in San Antonio, Texas, USA. A total of 360 mother-child pairs (90 pairs per center) are to be randomly and evenly allocated to either the intervention or the control group. Intervention group will receive four-session group obesity prevention education. Control group will receive a four-session personal and food hygiene education. The education is delivered by trained peer-mother promotoras. Data will be collected using questionnaires and focus groups. The primary outcome is a change in proportion of mothers with accurate MPCW. Secondary outcomes include change in maternal feeding styles and practices, maternal self-efficacy and actions for managing child excessive weight gain.

McNemar’s Test will be used to test the primary outcome. The GLM Univariate procedure will be used to determine intervention effects on secondary outcomes. The models will include the secondary outcome measures as the dependent variables, treatment condition (intervention/control) as the fixed factor, and confounding factors (e.g., mother’s education, children’s gender and age) as covariates. Sub-analyses will be performed to compare intervention effects on primary and secondary outcomes between the samples from Mexico and Texas, USA. Qualitative data will be analyzed through analysis of inductive content. A combined coding model will be developed and used to code transcripts using the NVivo software.

**Discussion:**

Healthy Change intervention could help change MPCW, an initial step for obesity prevention among preschoolers. This study presents a first of its kind intervention available in Spanish and English targeting Mexican and Mexican-American mothers in Mexico and USA.

**Trial registration:**

ISRCTN12281648

## Background

Childhood obesity is a worldwide epidemic that disproportionally affects certain racial/ethnic groups [1]. Mexico is among the countries with the highest prevalence of childhood obesity [2, 3]. Obesity is more prevalent in Mexican-American children than their white counterparts in the United States of America (USA) [4]. Childhood obesity has been found to increase health risks, e.g. hyperlipidemia, hypertension and abnormal tolerance to glucose. In addition, it has been documented that overweight (OW) preschoolers were four times more likely to become obese (OB) adolescents than their normal weight counterparts [4]. Given the consequences of overweight and obesity on physical and psychosocial health, as well as the heavy economic burden on health care, Public Health interventions are urgently needed to fight this epidemic in both USA and Mexico [5].

Obesity is a multi-faced etiological disease with risk factors such as genetic predisposition [6], increased energy consumption, sedentary lifestyle, poor socio-economic status, as well sociocultural factors and false beliefs about childhood obesity [7, 8]. Unlike adults, children cannot choose the environment in which they live or the food they eat [9]. Mothers are influential in shaping life habits of young children, thus important agents for childhood obesity prevention [10, 11]. However, research has shown that mothers of young children, do not perceive overweight or obesity as a health threat. Instead, they prefer chubby children [12, 13]. In Mexico, a study on mothers and children residing in 5 northeastern states found that 68% of mothers of overweight children and 75% of mothers of obese children underestimated their preschoolers’ body weight [14]. In addition, Mexican and Hispanic mothers exert control and discipline that could lead to over consumption of foods and increased obesity risk [15, 16]. More problematic, when mothers were not aware of their children’s overweight status, they had a lower self-efficacy to manage behaviors related to their child’s weight in comparison to mothers of normal weight children [17, 18]. Such research findings highlight the need for correcting mothers’ misperception of their children’s overweight status as the first step for obesity [8, 19, 20].

Childhood obesity prevention programs should incorporate strategies targeting mothers’ misperception of their children’s weight status. A study conducted in Iran documented the impact of an educational intervention on maternal perception of school children’s weight. The study showed a significant increase in the capacity of mothers to recognize their children’s obese weight status [21]. Interventions to prevent and treat OB in preschool Mexicans are scarce and have been directed to diet and exercise, showing little effect or none [22]. Also in these interventions, maternal perception of children’s weight has not been evaluated (MPCW) prior to incorporate participants in the intervention programs. As such, the “Healthy Change” intervention is designed to change maternal misperception of her child’s weight (MPCW) through peer-led parental education in childcare settings.

## Methods

### Aims

This pilot study aims to develop and test the effects of the “Healthy Change” intervention on changing maternal perception of preschoolers’ weight status among Mexican mothers in México, and Hispanic mothers in San Antonio, Texas. Specific objectives are 1) To test the feasibility of “Healthy Change” intervention when implemented in a childcare setting; 2) To assess the effect of “Healthy Change” intervention on changing maternal perception of their preschoolers’ weight status; and 3) To assess the effect of the “Healthy Change” intervention on changing parenting style, maternal attitude, self-efficacy and practice toward raising healthy weight children.

### Trial design

The “Healthy Change” study is a multicenter two-arm randomized trial with four research sites, three in Mexico: a) Nuevo León, b) Tamaulipas, and c) Zacatecas, and one in San Antonio, Texas, USA. At each site, two childcare centers are randomly assigned to either the intervention or the attention control group. A total of 360 pairs of preschoolers and their mothers, i.e., 90 pairs per site, are recruited for the trial. Intervention participants will receive a four-weekly group education focusing on maternal perception of children’s bodyweight that facilitate healthy behavioral changes for the family. Control participants will receive a four-session group education on food hygiene and first aids as attention control. The outcomes include change in the proportion of mothers who accurately estimate their children’s weight categories, change in feeding style, maternal self-efficacy and actions taken in managing the child’s excessive weight gain. Data are to be collected at baseline and the end of the four-week group education from both groups. The study began on 08–29-2015, and will end on 11–20-2018.

### Study setting

The study will be conducted in childcare settings, i.e., kindergarten classes in Mexico and Head Start Centers in Texas, USA.

### Sample size and statistical power

The primary outcome of this intervention will be the proportion of mothers who adequately estimate their child’s weight status. Assuming the intra-class correlation coefficient (ICC) of 0.03, to detect a change of mother’s accurate estimation of children’s weight status from 50 to 70% with 80% power, 37 mother-child pairs would be required per research center per arm [23]. Accounting for a 15% attrition rate, each research site will enroll 45 pairs of mothers and children for the intervention and another 45 pairs for the control group. The total sample size for this four-center trial will be 360 pairs of mothers and children.

### Childcare center recruitment and randomization

Each research site will recruit a total of 90 pairs of preschoolers and their mothers with 45 pairs in the intervention and 45 pairs in the control group. Each research site initially recruits two childcare institutions with similar size and demographic profiles to participate in the study. The administrators of these centers are informed of the possibility of being assigned to either the intervention or the control group. Prior to participant recruitment, the two or three childcare institutions at each research center are randomly assigned to one of the two treatment groups using a random number table. In the event that an insufficient sample is recruited from the participating centers, a third center will be added to ensure adequate sample in the designated group.

### Participant inclusion and exclusion criteria

Mothers of Mexican descent, along with their preschoolers between the age of 3 and 5 years old enrolled in participating childcare centers are eligible for the study. Preschoolers.

### Participant recruitment

Participants will be recruited through meetings, flyers, and take-home letters. Interested parents will be screened for study eligibility and informed consent will be obtained prior to data collection.

### Intervention

The intervention “Healthy Change” is informed by the Social Cognitive Theory (SCT) [24]. SCT describes human behavior as a dynamic and reciprocal interaction of personal factors, behavior, and the environment [24]. Based on the SCT, Healthy Change intervention strategies focus on cognitive and behavioral skills to enable mothers to take actions in managing their child’s weight. The application of Social Cognitive Theory Concepts is shown in Table 1. The Healthy Change intervention curriculum consists of four sessions: 1) Understanding excess weight and obesity as a health problem and current and future health consequences; 2) How is my child’s weight?; 3) Maternal feeding styles and physical activity; and 4) I can!, which includes strategies to management child’s health behaviors.

**Table 1:** Social Cognitive Theory (SCT) Concepts and Components of “Healthy Change” Intervention Group

Concepts of SCT	Intervention strategies
Reciprocal determinism: explore personal and environmental factors that can contribute to the change of cognition and behavior.	-Investigate the maternal beliefs related to OW-OB -Etiology of obesity: genetic factors and environmental -Present and future health effects of OW-OB -Recognize signs indicating that a child is OW-OB -Image perceived vs actual image
Self – efficacy: Increase Self-efficacy to manage child’s life style behaviors related to weight. Modeling - learning by observation	- Explore achievements that have been reached in management child’s life style behaviors related to weight and techniques which could be generalized as desired behaviors. - Ask the mother to comment an attempt which failed and explore individual and environmental factors that might have contributed in this attempt failed. - Teaching strategies of calm moment and time out. - Ask the mother that practice strategies and provide feedback
Behavioral capacity: providing opportunities for the mother to master necessary skills	- Together, identify actions that can be performed to manage child’s life style behaviors related to weight.
Reinforcement: Increase the expectations of outcomes’ behavior Reinforcing positive behaviors.	- Together, identify maternal feeding style and feeding practices of the participating mothers. - To identify factors of which contribute to the increase of child’s weight. - Teaching the participating mother practices that contributes to a healthy child’s weight.

Attention control is considered a valid control condition when conducting trials of social-behavioral interventions such as Healthy Change. We developed an attention control curriculum with a similar dose of group education entitled: *“Hygiene and Health Promotion”*. The four weekly group sessions cover the following topics: 1) The mother as her child’s hygiene promoter; 2) Hygiene and food safety at home; 3) Best ways to store food in your kitchen; and 4) Accident prevention at home and surrounding areas.

Each session for both groups will last about 90 min and will be held in a room within the participants’ own childcare institution. Both the Healthy Change and Hygiene and Health Promotion curriculum are available in two languages, Spanish and English. In Mexico, the content is delivered in Spanish. In the USA, mothers select the language of their preference for group education.

### Procedure to implement the intervention

The Healthy Change study uses a train-the-trainer model for intervention delivery. Each research site director is responsible for identifying and recruiting Promotoras from the participating childcare centers. To qualify, a Promotora must have a child enrolled at the participating childcare center. Promotora training includes 6 h of face-to-face training on the overall study, each session’s content, take home activities, and interpersonal communication skills. Promotoras take and must pass a certification exam in order to qualify for delivering the parent sessions. If she does not pass the exam, she will receive remediation and will be asked to retake the exam. Promotoras who do not pass the exam on the second attempt will not qualify to serve as Promotoras for the study.

The intervention includes Promotora-led sessions held in the classroom. Promotoras will use an English or Spanish facilitator guide to deliver the content. Promotoras have access to a projector and screen, whiteboard and markers or chalk, pencils, sheets and participant rosters.

### Evaluation methods

#### Process evaluation

Process evaluation of the intervention will include multiple data sources including, but not limited to, session duration, attendance, and context. Research personnel will observe a random group education session to assess delivery using a Healthy Change Fidelity Checklist. Focus groups will be conducted with up to 12 participating mothers in the intervention group at each research site at the end of the intervention. Program participants will be invited to participate in these group discussions on the day of the endpoint questionnaire. At least one focus group will be held at each participating center. An external experienced moderator along with a co-facilitator will use a semi-structured interview guide to facilitate each focus group. All focus group meetings will be audio-recorded and transcribed verbatim. The recordings will be transcribed and imported into NVIVO software for analysis [25].

#### Outcome evaluation

Baseline and post-intervention data will be collected on all participants. Each mother will receive 25 USD at the end of the intervention for their participation in the data collection. The primary outcome of this study is the proportion of mothers who accurately estimate their children’s weight status. Maternal perception of child’s weight is measured using the Parents’ Perceptions about Their Child’s Appearance and Health Questionnaire [19]. Mothers are asked to indicate their child’s weight status from 5 response options: “underweight”, “a little underweight”, “about the right weight”, “a little overweight”, or “overweight” (MPCW by words). Mothers also checked one of the body sketches (MPCW by image) in the age range 2 to 5 years old [19, 26].

Secondary outcomes include:

- Change in the maternal feeding styles. Maternal feeding styles is measured using the Caregiver Feeding Style Questionnaire. The questionnaire consists of 19 questions grouped in two dimensions: demandingness and responsiveness. Subsequent calculation of the median of both dimensions places participants in one of the four styles, i.e., authoritative, authoritarian, indulgent, or uninvolved [16].
- Increased self-efficacy score of maternal influencing on children’s health behavior. Mothers rate their confidence on a 5-point Likert scale ranging from “strongly disagree” to “strongly agree” on these two statements: “I can influence my child’s food choices” and “I can influence my child’s amount physical activity” [27].
- Improved score of family nutrition and physical activity. The Family and Nutrition and Physical Activity (FNPA) screening tool is used to assess mothers’ practices that may predispose children to becoming overweight [28]. The FNPA tool consists of 20 questions assessing family meals, family eating practice, food and beverage choices, material feeding restriction/ reward practice, child’s screen and activity time, sleep schedule, family activity routine and healthy environment [28]. Each question has four options: i.e., “Never/Almost Never”, “Sometimes”, “Often”, and “Very Often/Always” [28].
- Reduction or maintenance of the child’s BMI and BFP: The child’s weight is measured by Seca Scale 813 and height by Seca stadiometer 214, the BFP is measured by bioelectrical impedance by InBody 230.

Maternal BMI is assessed as a medicating factor. The maternal weight is measured using Seca scale 813 and the height with stadiometer SECA 214. Subsequently, the maternal BMI is calculated and classified as: low weight (< 18.5), normal weight (18.5–24.9), pre-OB (25.0 to 29.9), OB I (30.0–34.9), OB II (35.0–39.9) and OB III (> 40) [29]. Maternal body fat percentage (BFP) will be measured by bioelectrical impedance by InBody 230.

A self-administered questionnaire is used to collect socio-demographic data, e.g., age, education level, marital status, monthly family income and child’s age and sex, as well as degree of acculturation using the Scale of Acculturation of the Hispanic for participants in Texas, USA [30].

### Data analysis

#### Healthy change intervention feasibility

Researchers will analyze qualitative data from the focus groups using inductive content analysis [25]. A team approach will be utilized to analyze data with a minimum of three researchers independently reviewing each transcript and identifying emerging themes. The team will then convene to discuss, compare, and merge themes to develop a coding template. NVivo software will be used to assist in data organization.

#### Healthy change intervention effect

The quantitative data will be analyzed in Statistical Package for the Social Sciences (SPSS) version 23. WHO Anthro (version 3.2.2, January 2011) and macros will be used to calculate children’s age- and gender- specific BMI percentile and children will be classified by the World Health Organization | BMI-for-age chart: malnutrition (percentile < 3), low weight (≥3 and < 15), normal weight (≥15 and < 85), OW (≥85 but < 97), and OB (≥97) [31]. The rate at which mothers accurately classify their children’s weight status will be calculated in two ways, i.e., by words or by images. Accurate MPCW by words will be defined as show in Table 2.

**Table 2:** Accurate MPCW by words

Children’s actual weight status by BMI percentile	MPCW by words
Malnutrition (< 3 percentile) Low weight (≥ 3 and < 15 percentile)	“underweight” or “a little underweight”
Normal weight (≥ 15 and < 85 percentile),	“Normal weight”
Overweight (≥ 85 but < 97 percentile)	“A little overweight”
Obese (≥ 97)	“Overweight”

With regard to the MPCW by image test, an accurate perception is defined when mothers of underweight and normal weight children select an image smaller than the average image or when mothers of children with OW-OB select the average or a larger image.

McNemar’s Test will be used to test the primary outcome measure, i.e., intervention effect on change in proportion of mother’s accurate recognition of their preschooler’s weight status. The GLM Univariate procedure will be used to determine intervention effects on secondary outcomes e.g., scores of mother’s self-efficacy, feeding practices and children’s BMI percentiles and fat%. The models will include the secondary outcome measures as the dependent variables, treatment condition (intervention versus control) as the fixed factor, and confounding factors (e.g., mother’s education level, children’s gender and age) as covariates. Sub-analyses will be performed to compare intervention effects on both primary and secondary outcomes between the samples from Mexico and Texas, USA.

### Ethics and dissemination

This project has been reviewed, approved and registered by Ethical Research Committee of College of Nursing of the Autonomous University of Nuevo León (FAEN-P-1144) and for The University of Texas at San Antonio, Institutional Review Board (16-203 N). Written informed consent to participate in the study was obtained from participants, and the mothers’ consent for the participation of their children.

All data will be stored in a secured place with access limited to research staff only. All hard copy files will be stored in locked filing cabinets within the laboratory and all electronic files will be stored on password-protected computers. Only the research staff will have access to files and the link connecting names to the issued identity codes. All laboratory staff will undergo rigorous training on data collection, data entry, and data storage procedures to ensure all participant information is protected.

Research records will not be released without a participant consent unless required by law or a court order. The data from participants may be used in publish and/or presentations, but participants’ identities will not be disclosed. The information discussed in focus groups, will be audiotaped, and transcribed by a trained research staff for the analysis. All storage of data will take place on password-protected computers that only research staff have access. No information to identify individuals or any institution will be reported. Participant identity, comments, as well as all audiotapes and written records will be kept confidential and secure. All data collected from these focus groups will have names removed upon collection, such that names will not be used at any time during the research process, or the dissemination findings. Following completion of the analysis, all tape recordings will be deleted. Research findings will be published in peer-reviewed journals and communicated to key audiences, including personnel of participants’ childcare centers.

## Discussion

Childhood OB is one of the most serious public health issues of the twenty-first century. Children with OB are at increased risk of obesity as adults and are more likely to develop chronic health conditions, such as diabetes and cardiovascular disease, at a younger age. It is therefore necessary to implement strategies from childhood to prevent and treat OW-OB paying special attention to parents, given that they not only act as behavior moderators, but also are responsible for the quality and quantity of food made available to their children. When parents are involved in interventions to prevent and treat weight problems in their children, there are more possibilities of achieving satisfactory outcomes.

The pilot study aims at correcting Mexican mothers’ misperception of their children’s weight status as the initial step of obesity prevention. Studies conducted in countries such as the USA, Italy, United Kingdom, Germany and Malaysia have assessed MPCW and found that the majority of mothers of children with OW-OB have misperceptions of their child’s weight, and they underestimate it; this misperception could have impact in other parenting strategies related to feeding and physical activity of their child. On the other hand, only one study conducted in Iran documented the impact of an educational intervention on maternal perception of her child’s OB in scholar age; therefore, we conduct this research to evaluate the feasibility and effect of “Healthy Change” intervention on MPCW of her pre-school child, through accuracy MPCW post intervention.

This collaborative research endeavor can produce promising outcomes, enabling the team to secure extramural funding for a large-scale randomized controlled trial. The innovative research inquiry will advance our knowledge of employing effective culturally appropriate prevention strategies to curb the childhood obesity epidemic facing both the United States of America and Mexico.

## Abbreviations

BFP: Body fat percentage
BMI: Body mass index
FNPA: Family Nutrition and Physical Activity
ICC: Intra-class Correlation Coefficient
ICC: Intra-class correlation coefficient
MPCW: Maternal perception of her child’s weight
OB: Obesity
OW: Overweight
SCT: Social Cognitive Theory
USD: United States dollar
